# Cigarette Smoking Exacerbates the Adverse Effects of Age and Metabolic Syndrome on Subclinical Atherosclerosis: The Bogalusa Heart Study

**DOI:** 10.1371/journal.pone.0096368

**Published:** 2014-05-02

**Authors:** Shengxu Li, Miaoying Yun, Camilo Fernandez, Jihua Xu, Sathanur R. Srinivasan, Wei Chen, Gerald S. Berenson

**Affiliations:** 1 Tulane Center for Cardiovascular Health and Department of Epidemiology, Tulane University School of Public Health and Tropical Medicine, New Orleans, Louisiana, United States of America; 2 College of Life and Environment Sciences, Minzu University of China, Beijing, China; Shanghai Institute of Hypertension, China

## Abstract

Age and metabolic syndrome are major risk factors for atherosclerosis. However, limited information is available regarding whether cigarette smoking, another major, modifiable risk factor, has synergistic effects with age and metabolic syndrome on subclinical atherosclerosis, particularly in young adults. This aspect was examined in 1,051 adults (747 whites and 304 blacks; aged 24–43 years) from the Bogalusa Heart Study. General linear models were used to examine the effects of cigarette smoking and its interactive effects with age and metabolic syndrome on carotid intima-media thickness (CIMT). After adjusting for age, race, and sex, current smokers had lower BMI (mean±SE: 27.4±0.4, 29.3±0.5, and 29.9±0.3 kg/m^2^ in current, former, and never smokers, respectively; p<0.0001) and lower levels of fasting glucose (82.8±0.9, 89.5±2.3, and 87.1±1.1 mg/dL, respectively; p = 0.001) and insulin (10.6±0.4, 14.2±1.0, 13.6±0. 6 µU/ml, respectively; p<0.0001). Despite being lean and having favorable levels of glucose and insulin, current smokers had greater CIMT (0.850±0.012, 0.808±0.011, and 0.801±0.006 mm, respectively; p = 0.0004). Importantly, cigarette smoking showed significant interactions with age and metabolic syndrome on CIMT: Age-related change in CIMT in current smokers was significantly greater (0.013±0.002 mm/year) than in nonsmokers (former and never smokers combined) (0.008±0.001 mm/year) (p for interaction = 0.005); the difference in CIMT between those with and without metabolic syndrome was significantly greater in current smokers (0.154±0.030 mm, p<0.0001) than in nonsmokers (0.031±0.014 mm, p = 0.03) (p for interaction<0.0001). In conclusion, cigarette smoking significantly exacerbates the adverse effects of age and metabolic syndrome on subclinical atherosclerosis in young adults, which underscores the importance of prevention and cessation of cigarette smoking behavior in the young.

## Introduction

Atherosclerotic diseases, like coronary heart disease, are leading causes of deaths[1]. Many risk factors contribute to the acceleration of these conditions[2]. And among the risk factors, age is the most important[3], [4]. Metabolic syndrome, a common cluster of interrelated cardiometabolic factors including obesity, insulin resistance, dyslipidemia, and elevated blood pressure, is also predictive of atherosclerotic diseases and related mortality[5]–[8]. Additionally, cigarette smoking is a well-established, powerful risk factor for morbidity and mortality associated atherosclerosis[9]–[15]_ENREF_8_ENREF_8. These risk factors often exert a joint effect on the development of atherosclerosis. For example, Framingham Risk Score, which combines multiple risk factors, is used for assessment of cardiovascular risk[16]–[19].

Although the joint effect of multiple risk factors has been well established, limited information is available regarding whether cigarette smoking, a very common and modifiable risk factor[20], strengthens the adverse effort of age and metabolic syndrome on atherosclerotic diseases. Such information is important in terms of early prevention and identification of individuals with cardiovascular risk. Carotid intima-media thickness (CIMT) measured by ultrasound is widely used as a valid noninvasive measurement for subclinical atherosclerotic diseases, and is linked to the presence, extent, and development of atherosclerosis[21]–[24]_ENREF_18. Few studies have examined whether the association of age and metabolic syndrome with CIMT is modified by the status of cigarette smoking[13]. In the current study, we tested the hypothesis that cigarette smoking exacerbates the effects of age and metabolic syndrome on CIMT in a community-based sample of young adults from the Bogalusa Heart Study.

## Methods

### Ethics statement

Written informed consent was obtained from each participant of the study. The protocol was approved by the Institutional Review Board of the Tulane University Health Sciences Center.

### Study sample

During 2000–2002, 1,203 young adults aged 24–43 years residing in a black-white community (65% white and 35% black) of Bogalusa, LA, were examined for cardiovascular risk factors[25]. Among these, 1,051individuals who had CIMT measurements were included in the current analysis. The cardiovascular risk factor variables included body mass index (BMI), waist circumference, low-density lipoprotein (LDL) cholesterol, high-density lipoprotein (HDL) cholesterol, triglycerides, fasting glucose and insulin, systolic and diastolic blood pressure, and cigarette smoking status, along with demographic and lifestyle variables.

### Examinations

The survey followed essentially the same protocol for risk factor measurements[26]. Participants were instructed to fast for 12 h before screening, with time and compliance ascertained by interview on the morning of the examination. Height, weight, and waist circumference were measured twice and the mean values were used. Information on cigarette smoking status, leisure time physical activity, alcohol intake, and medications for type 2 diabetes, hypertension, and high cholesterol was obtained as part of a health habit questionnaire[27]. For cigarette smoking status, participants were grouped into three categories: current smokers, former smokers, and never smokers. Current smoking was defined by “I smoke at least one cigarette a week”; former smoking was defined by “I used to smoke at least one cigarette a week”; and never smoking was defined as not being a current or a former smoker. Alcohol drinking was defined by “drink alcohol during the past 12 months”. Leisure time physical activity was categorized into three groups, low, moderate, and vigorous physical activity, according to a grade of 1–5 for physical activity outside of work, with 1 being most inactive and 5 most active (1 = low; 2–3 = moderate; 4–5 = vigorous). Replicate blood pressure measurements were obtained on the right arm of the participants in a relaxed, sitting position. Arm measurements, length and circumference, were made during the examination to ensure the use of a proper cuff size. Systolic and diastolic blood pressure levels were recorded as the first and fifth Korotkoff phases using mercury sphygmomanometers. These blood pressure readings were taken by each of two randomly assigned and trained observers and levels were reported as the mean of six replicate readings.

### Laboratory analyses

Serum total cholesterol and triglycerides levels were measured by enzymatic procedures on the Hitachi 902 Automatic Analyzer (Roche Diagnostics, Indianapolis, IN). Serum lipoprotein cholesterols were analyzed using a combination of heparin–calcium precipitation and agar–agarose gel electrophoresis procedures[28]. The laboratory has been monitored for precision and accuracy of lipid measurements by the Lipid Standardization and Surveillance Program of the Centers for Disease Control and Prevention, Atlanta, GA. A commercial radioimmunoassay kit was used for measuring plasma immunoreactive insulin levels (Pharmacia Diagnostics, Piscataway, NJ). Plasma glucose levels were measured as part of a multiple chemistry (SMA20) profile by a glucose oxidase method.

### Carotid ultrasonography

Trained sonographers performed ultrasound examinations with a Toshiba Sonolayer SSH160A, a 7.5 MHz linear array transducer on subjects in the supine position with the head slightly extended and turned to the opposite direction of the carotid artery being studied. Images were recorded at the common carotid, carotid bulb (bifurcation), and internal carotid arteries bilaterally according to previously developed protocols for the Atherosclerosis Risk In Communities Study[29]. Images were recorded on S-VHS tapes and read by certified readers from the Division of Vascular Ultrasound Research using a semiautomatic ultrasound image processing program developed by the California Institute of Technology Jet Propulsion Laboratory (Pasadena, California) according to strict protocols[29], [30]. The mean of the maximum carotid IMT readings of three right and three left far walls for common, bulb and internal segments was used. Subjects who had missing values on both sides of any of the three sites were excluded from the current study.

### Statistical methods

Data analyses were performed using SAS 9.3. CIMT was used as the outcome variable. Diagnostic criteria for components of metabolic syndrome from the updated NCEP ATP III guidelines[31] included: (1) abdominal obesity (waist circumference ≥102 cm for men and ≥88 cm for women), (2) hypertriglyceridemia (fasting triglycerides≥150 mg/dl), (3) low HDL cholesterol (<40 mg/dl for men and <50 mg/dl for women, or taking cholesterol lowing mediction), (4) high blood pressure (≥130/≥85 mmHg or taking antihypertensive medication), and (5) high fasting glucose (≥100 mg/dl or taking antidiabetic medication). CIMT, waist circumference, triglycerides, and insulin were log-transformed to improved normality before analytical comparisons. Chi-square test was used to examine differences in proportions of dichotomous variables in different study groups. General linear models were used to examine the associations between risk factor variables and CIMT. To examine the interaction effects of cigarette smoking with age and metabolic syndrome, interaction terms between these variables were included in the model with the main terms included as well in a single model. After significant interactions between age and smoking and between metabolic syndrome had been identified, we performed stratified analyses by cigarette smoking status while adjusted for race, sex, leisure time physical activity, alcohol intake, LDL cholesterol, insulin, medications for type 2 diabetes, hypertension, and high cholesterol, and metabolic syndrome or age, when appropriate. We also performed sensitivity analyses stratified by race and sex.

## Results

Overall, 30.7% (323/1051) of the participants were current smokers, and 20.7% (220/1051) had metabolic syndrome. Blacks vs. whites and females vs. males had favorable lipids levels; blacks vs. whites and males vs. females had higher blood pressure and fasting glucose levels and greater CIMT (**Table 1**).

**Table 1:** Characteristics of the study sample by race and sex: The Bogalusa Heart Study.

Study variable	White		Black		P for difference	
s	Male (n = 336)	Female (n = 411)	Male (n = 116)	Female (n = 188)	Race	Sex
Age (years)	36.6±4.4	36.4±4.4	36.7±4.3	35.5±4.9	0.12	0.09
Cigarette smoking					0.01	0.63
Current smokers (%)	29.8	28.7	39.7	31.4		
Former smokers (%)	13.7	14.6	7.8	8.0		
Never smokers (%)	56.5	56.7	52.6	60.6		
Leisure time physical activity					0.005	<0.001†
Low (%)	7.1	12.2	13.8	14.9		
Moderate (%)	56.3	64.7	47.4	52.1		
Vigorous (%)	36.6	23.1	38.8	33.0		
Alcohol drinking (%)	71.4	60.6	76.7	51.6	0.04‡	<0.001
Metabolic Syndrome (%)	24.7	17.3	23.3	20.7	0.69	0.01†
Diabetes medication (%)	3.3	1.5	3.5	3.2	0.35	0.18
Antihypertensive medication (%)	8.3	3.9	10.3	10.6	0.001‡	0.01†
High cholesterol medication (%)	4.2	2.9	1.7	1.1	0.06	0.25
BMI (kg/m^2^)	29.0±5.6	28.2±7.1	29.3±6.9	30.9±8.0	<0.001‡	0.07
Waist circumference (cm)	99.0±14.5	86.8±16.3	96.9±17.1	93.0±17.8	<0.001‡	<0.001†
LDL Cholesterol (mg/dL)	129.8±34.3	124.0±32.7	125.5±41.6	114.4±30.5	0.002	0.001
HDL Cholesterol (mg/dL)	41.2±12.1	50.6±13.0	48.9±15.5	52.2±13.4	<0.001¶	<0.001
Triglycerides (mg/dL)	163.0±129.5	121.9±70.1	132.4±112.0	89.0±40.3	<0.001	<0.001
Glucose (mg/dL)	87.9±21.6	82.4±14.6	91.1±34.1	87.9±32.3	0.002	0.002
Insulin (µU/ml)	13.1±10.1	11.3±7.9	12.3±9.8	15.5±20.5	<0.001‡	<0.02£
Systolic BP (mm Hg)	118.2±10.9	111.0±11.2	128.4±16.5	118.8±16.0	<0.001	<0.001
Diastolic BP (mm Hg)	74.8±8.2	69.6±8.8	80.8±12.8	73.9±11.5	<0.001	<0.001
CIMT (mm)	0.861±0.190	0.763±0.125	0.898±0.217	0.804±0.160	<0.001	<0.001

Mean±SDs are shown unless otherwise noted.

LDL: low-density lipoprotein; HDL: high-density lipoprotein; BP = Blood pressure; CIMT: carotid intima-media thickness.

† : only in whites;

‡ : only in females;

¶ : only in males;

£ : in opposite direction in different race groups.

P values were adjusted for age and race/sex.

Current smokers had lower BMI, smaller waist circumference and lower levels of fasting glucose and insulin than nonsmokers (**Table 2**). Despite being leaner and having improved insulin resistance, current smokers had greater CIMT than nonsmokers (least square mean±SE: 0.853±0.009, 0.796±0.013 and 0.801±0.006 mm in current, former, and never smokers, respectively; p<0.0001, adjusted for race, sex, leisure time physical activity, alcohol intake, LDL cholesterol, insulin, medications for type 2 diabetes, hypertension, and high cholesterol, LDL cholesterol, insulin, and metabolic syndrome); however, there was no significant difference in CIMT between former and never smokers (p = 0.72). After similar adjustment, age was significantly associated with CIMT, with an increase of 0.010±0.001 mm (β±SE) in CIMT each year (p<0.0001); metabolic syndrome remained significantly associated CIMT, with an increase of 0.062±0.014 mm in CIMT (p<0.001) for those with metabolic syndrome compared to those without.

**Table 2:** Risk factor variables by cigarette smoking status: The Bogalusa Heart Study.

Risk factor variables	Never smokers (n = 598)	Former Smokers (n = 130)	Current Smokers (n = 323)	P value
Age (years)	36.1±0.2	37.5±0.4	36.4±0.2	0.006
BMI (kg/m^2^)	29.9±0.3	29.3±0.5	27.4±0.4	<0.0001
Waist circumference (cm)	94.4±0.7	93.8±1.4	90.0±0.9	<0.0001
Metabolic syndrome (%)	20.6	26.2	19.5	0.27
Diabetes medication (%)	2.0	5.4	2.5	0.09
Antihypertensive medication (%)	7.4	9.2	6.2	0.52
High cholesterol medication (%)	2.7	5.4	2.2	0.16
LDL Cholesterol (mg/dL)	125.5±1.4	126.2±3.0	121.4±1.9	0.19
HDL Cholesterol (mg/dL)	48.1±0.5	48.1±1.2	46.6±0.8	0.22
Triglycerides (mg/dL)	127.7±4.0	142.0±10.9	130.5±4.9	0.27
Glucose (mg/dL)	87.1±1.1	89.6±2.3	82.8±0.9	0.002
Insulin (µU/ml)	13.6±0.6	14.2±1.0	10.6±0.4	<0.0001
Systolic BP (mm Hg)	115.9±0.5	116.6±1.2	118.0±0.8	0.26
Diastolic BP (mm Hg)	73.2±0.4	73.2±0.9	73.5±0.6	0.94
CIMT (mm)	0.801±0.006	0.808±0.011	0.850±0.012	0.002

Mean±SEs are shown unless otherwise indicated.

LDL: low-density lipoprotein; HDL: high-density lipoprotein; BP: Blood pressure; CIMT: carotid intima-media thickness.

P values were adjusted for race and sex (and age).

**Figure 1:**
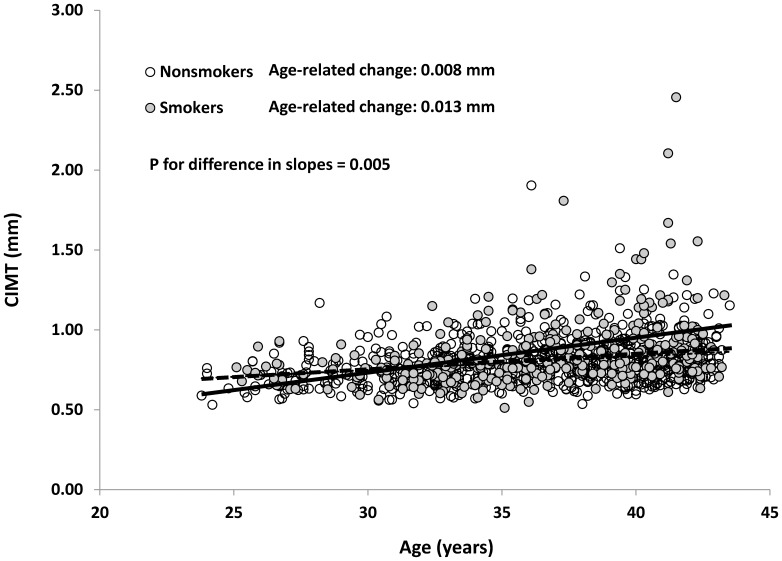
Scatter plot between age and carotid intima-media thickness (CIMT) in cigarette smokers and nonsmokers. P value was adjusted for race, sex, leisure time physical activity, alcohol intake, LDL cholesterol, insulin, medications for type 2 diabetes, hypertension, and high cholesterol, LDL cholesterol, insulin, and metabolic syndrome components.

**Figure 2:**
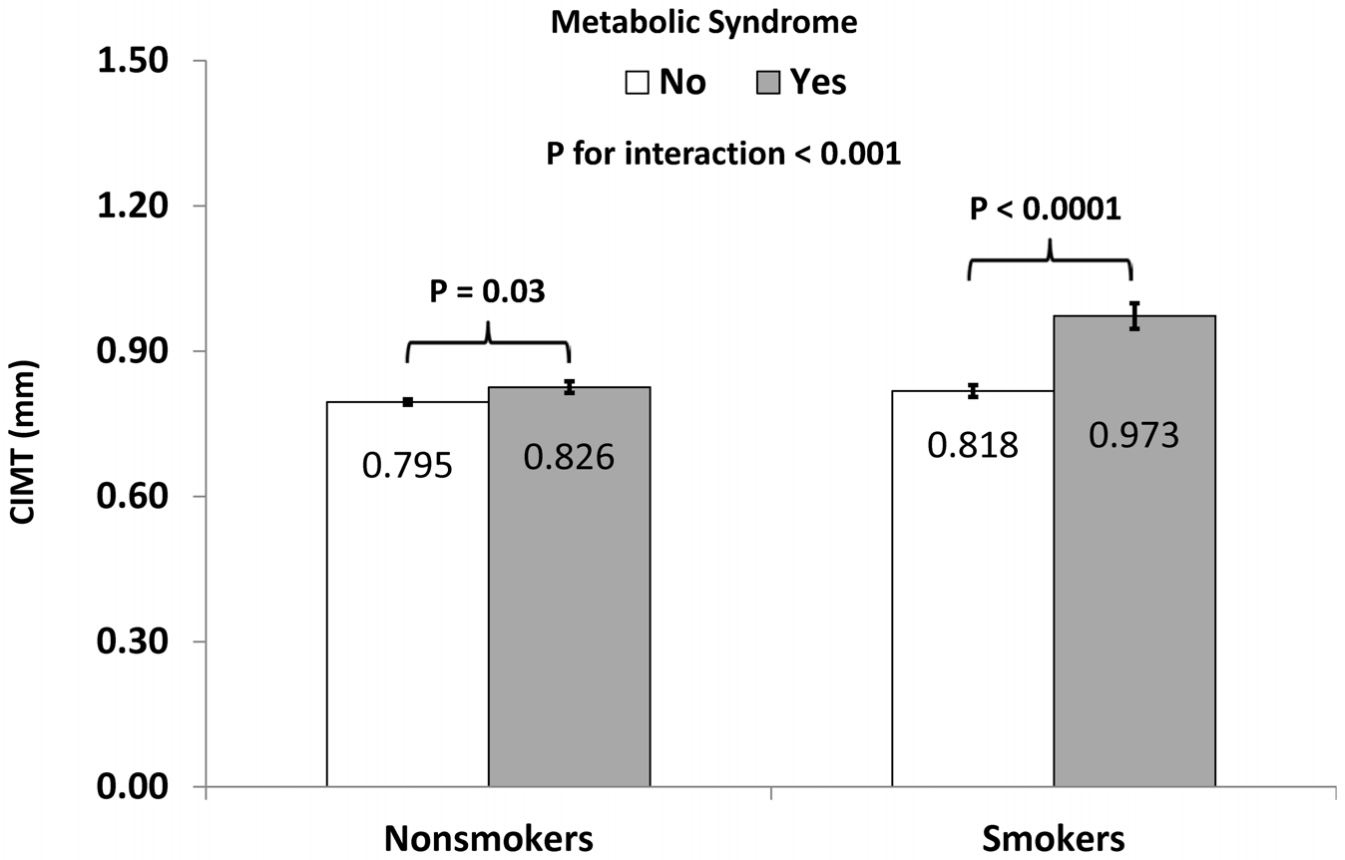
Carotid intima-media thickness (CIMT) by cigarette smoking status and metabolic syndrome. Metabolic syndrome was defined according to the updated NCEP ATP III guidelines[31]. P value was adjusted for race, sex, leisure time physical activity, alcohol intake, LDL cholesterol, insulin, medications for type 2 diabetes, hypertension, and high cholesterol, LDL cholesterol, insulin, and metabolic syndrome components.

Smoking significantly modified the associations between age and CIMT (p for interaction  =  0.03) and between metabolic syndrome and CIMT (p for interaction  =  0.001). Because there was no difference in this modifying effect between never smokers and former smokers (p for interaction with age  =  0.57 and p for interaction with metabolic syndrome  =  0.13) and there was no significant difference in CIMT between never smokers and former smokers, we combined former smokers and never smokers as nonsmokers. As shown in **Figure 1**, age-related change in CIMT was 0.008±0.001 mm/year in nonsmokers (p<0.0001) and 0.013±0.002 mm/year in current smokers (p<0.0001) (p for interaction = 0.005). In **Figure 2**, the difference in CIMT between those with metabolic syndrome and those without was significantly greater in smokers (0.973±0.026 vs. 0.818±0.012 mm; difference 0.154±0.030 mm, p<0.0001) than in nonsmokers (0.826±0.012 mm vs. 0.795±0.006 mm; difference 0.031±0.014 mm, p = 0.03) (p for interaction<0.0001). The observed interactions remained significant when we further adjusted for internal diameter at diastole (p = 0.04 for interaction with age and p = 0.0002 for interaction with metabolic syndrome).

For race and sex stratified analyses, overall similar trends for interactions between age and cigarette smoking and between metabolic syndrome and cigarette smoking were observed as in the total sample, though the degree of significance of the interaction terms varied (p = 0.09 and p = 0.04 in white males; p = 0.94 and p = 0.007 in white females; p = 0.06 and p = 0.15 in black males; and p = 0.18 and p = 0.006 in black females, for interaction with age and for interaction with metabolic syndrome, respectively).

## Discussion

In the current study, cigarette smokers had significantly higher CIMT than nonsmokers despite having lower BMI and waist circumference and improved insulin resistance. If CIMT represents vascular age for an individual, current smokers were about 6–7 years older than nonsmokers in terms of their respective vascular ages (0.052–0.057 mm difference in CIMT between current smokers and nonsmokers divided by age-related change 0.008 mm/year in nonsmokers). Further, cigarette smoking exacerbated the adverse effects of age and metabolic syndrome on CIMT so that the influence of age and metabolic syndrome on CIMT was much stronger in current smokers than in nonsmokers. These findings from a community-based sample emphasize the importance of prevention and cessation of cigarette smoking behavior in the young.

Current smokers have a smaller overall body size reflected in lower BMI and smaller waist circumference than nonsmokers. This is consistent with previous reports[32], [33]. Further, smokers had lower levels of insulin and glucose than nonsmokers, independent of obesity measures such as BMI or waist circumference. The relationship between cigarette smoking and insulin resistance has not been consistent across studies. Henkin et al. did not find any association between active smoking and insulin sensitivity in the Insulin Resistance Atherosclerosis Study[34]. Similar findings were reported by Hughes et al.[35]. However, in a large prospective study, Cho et al. reported that smoking was an independent risk factor for type 2 diabetes, particularly among those with low insulin secretion and high insulin resistance at baseline[36]. Onat et al. found in a longitudinal study that smoking in males led to reduced insulin sensitivity and in females to increased insulin sensitivity[37]. The reasons for such disagreement among studies are not clear. Influences of a combination of some unaccounted for socioeconomic and/or lifestyle factors and potential biases may contribute to such discrepancies.

Despite being leaner and having improved insulin resistance, cigarette smokers had greater CIMT than nonsmokers, indicating that these cigarette smokers had increased atherosclerosis. Apparently, the adverse effects of cigarette smoking overtook the potential benefits, if any, of decreased obesity and low insulin resistance due to cigarette smoking. We have previously shown cigarette smokers have lower small artery compliance and higher systemic vascular resistance than nonsmokers[38]. Our study is also consistent with the findings from the Cardiovascular Risk in the Young Finns study[39], [40] and from a Chinese study[9]. In addition to its direct effect on CIMT, cigarette smoking also strengthened the adverse effects of age and metabolic syndrome, two major determinants of atherosclerosis, on the vasculature. Age-related change in CIMT in nonsmokers was only 0.008 mm, which increased to 0.013 mm in current smokers. As a result, it can be easily inferred that cigarette smokers not only have increased CIMT, but also have accelerated vascular aging process, which at least partially explains why cigarette smokers have increased risk for cardiovascular events. Further, the adverse effect of metabolic syndrome on CIMT was vastly increased from 0.031 mm in nonsmokers to 0.154 mm in current smokers. Such difference emphasizes the synergistic effects between cigarette smoking and metabolic syndrome and between cigarette smoking and age on the vasculature of the young.

Several mechanisms may explain the increased burden of atherosclerosis in current cigarette smokers. Cigarette smoking is known to have a deleterious effect on endothelial cells and thus increased invasion of lipids particles and monocytes[15]. Secondly, cigarette smoking also increases inflammation, which is an important initiator and contributor to atherosclerosis[15]. Several inflammation markers, like C-reactive protein, interleukin-6, and tumor necrosis factor alpha, along with adhesion molecules may be involved[15]. In addition, there are more than 4,000 known chemicals in cigarette smoke, many of which may have potential damaging effects on the vasculature[15]. Finally, age and metabolic syndrome are also linked to endothelial dysfunction, inflammation, and oxidative stress[41]. Deleterious effects by aging and metabolic syndrome may start and maintain a vicious cycle that significantly accelerates atherosclerosis[41], [42]_ENREF_41, as seen in our study from a sample of relatively young adults. Our study suggests that this vicious cycle is strengthened by the adverse effects of cigarette smoking.

Results of our study strongly support prevention of cigarette smoking behavior in young adults. As indicated by the greater CIMT among current smokers, cigarette smokers at an average age of 36.4 years already had increased atherosclerosis, which may be very difficult to reverse. In addition, the adverse effect of metabolic syndrome was less significant in nonsmokers. All these results argue for effective health education programs to prevent cigarette smoking at a young age. Moreover, results from our study also emphasize the importance of smoking cessation on vascular health because cigarette smoking significantly accelerated the age-related change in CIMT. It is also important to target cigarette smokers with metabolic syndrome.

The current study has important strengths. Our study was community-based, which makes our results more generalizable to a larger population with a similar demographic composition. In addition, data collection process followed a strict protocol with effective quality assurance/control measures. Finally, instruments to measure cigarette smoking and CIMT have been previously validated. On the other hand, our study was cross-sectional in nature. Potential confounding and selection bias might be present. However, since participants were not aware of their CIMT measurements, the observed associations were less likely to be distorted as a result of changed behavior or reverse causation. Nevertheless, longitudinal studies are needed to further confirm the results in the current study. We also recognize that the usefulness and definition of metabolic syndrome remains controversial[43], [44]. The interaction between cigarette smoking and metabolic syndrome may have reflected interactions between cigarette smoking and individual components of metabolic syndrome. Future studies should examine whether the interaction between cigarette smoking and metabolic syndrome is driven by interactions with certain individual components of metabolic syndrome. Finally, it should be noted that we used a composite value derived from measurements in different carotid sites. Due to different hemodynamic and anatomic properties in these sites, interactions observed in the current study may vary according to site, which warrants site-specific analysis in future studies.

In conclusion, the current study demonstrates that cigarette smoking is associated with increased atherosclerosis as indicated by CIMT. Further, cigarette smoking exacerbates the adverse effects of age and metabolic syndrome on the vasculature. Our results emphasize the importance of prevention and cessation of cigarette smoking behavior in the young. Future longitudinal studies are needed to confirm the current findings and to evaluate the effects of cessation of cigarette smoking behavior.
